# Combined Effects of Dietary Astaxanthin and β-Carotene on Antioxidant Status, Pigmentation, Muscle Quality, and Flavor Profile in Male and Female *Macrobrachium rosenbergii*

**DOI:** 10.3390/antiox15060711

**Published:** 2026-06-03

**Authors:** Zhouyu Chen, Jianlin Guo, Shunxiao Shi, Pengyuan Zhang, Yansong Xue, Yucai Xue, Bin Han, Kelvin Zhao Kang Ong, Zhixiao Ma, Weidong Yang, Xinjun Gang, Yanzi Liang, Yuhan Guo, Taranat Jiasalati, Amina Moss, Xuxiong Huang, Yukun Zhang, Weilong Wang

**Affiliations:** 1 China-ASEAN Belt and Road Joint Laboratory on Mariculture Technology, Shanghai 201306, China; 2 Key Laboratory of Healthy Freshwater Aquaculture, Ministry of Agriculture and Rural Affairs, Zhejiang Institute of Freshwater Fisheries (Zhejiang Freshwater Fishery Environmental Monitoring Station), Huzhou 313001, China; 3 Institute of Aquaculture, University of Stirling, Stirling FK94LA, UK; 4 Centre for Research on Environmental Ecology and Fish Nutrition of the Ministry of Agriculture, Shanghai 201306, China; 5 National Demonstration Center for Experimental Fisheries Science Education, Shanghai Ocean University, Shanghai 201306, China; 6 Laboratory of Aquatic Animal Nutrition, Faculty of Fisheries, Kagoshima University, Kagoshima 890-0056, Japan

**Keywords:** astaxanthin, β-carotene, *Macrobrachium rosenbergii*, antioxidant status, edible quality, sex-specific response, diet trail

## Abstract

Carotenoid nutrition plays a crucial role in crustacean aquaculture by regulating antioxidant defense, pigmentation, physiological performance, and edible quality. This study evaluated the combined effects of dietary astaxanthin and β-carotene on antioxidant status, pigmentation, muscle quality, and flavor profile in male and female *Macrobrachium rosenbergii*. Seven isonitrogenous and isolipidic diets were formulated, including a carotenoid-free control and six diets supplemented with different astaxanthin and β-carotene combinations. After a 77-day feeding trial, growth performance, antioxidant parameters, digestive enzyme activities, carotenoid deposition, body coloration, muscle texture, free amino acids, flavor nucleotides, equivalent umami concentration, and volatile flavor compounds were systematically assessed. Dietary carotenoid supplementation improved growth performance in both sexes, although the response patterns differed between males and females. In males, astaxanthin contributed more prominently to growth and several physiological traits, whereas in females, astaxanthin, β-carotene, and their interaction significantly affected multiple response variables. Carotenoid supplementation enhanced antioxidant capacity, carotenoid deposition, body redness, muscle texture, and flavor-related traits. GC-IMS analysis further revealed sex-dependent remodeling of volatile flavor profiles under different carotenoid combinations. Among all treatments, the combined high-dose diet containing 160 mg/kg astaxanthin and 160 mg/kg β-carotene showed the best overall performance in both sexes. These findings indicate that dietary astaxanthin and β-carotene combinations exert compound- and sex-dependent effects in *M. rosenbergii* and provide a basis for developing sex-specific functional feeds.

## 1. Introduction

The giant freshwater prawn, *Macrobrachium rosenbergii*, is one of the most economically important freshwater crustacean species in global aquaculture [1]. However, intensive farming conditions frequently disturb systemic redox homeostasis and induce oxidative stress, which can accelerate lipid peroxidation, impair growth and immune competence, and damage muscle structure, ultimately reducing edible quality and flavor value [2,3]. In commercial production, these oxidative challenges have become a major constraint on both biological performance and product quality.

In addition to oxidative stress, *M. rosenbergii* is characterized by pronounced sexual dimorphism. Males generally grow faster than females because of differences in metabolism, endocrine regulation, and energy allocation [4]. These sex-specific traits have encouraged the development of sex-segregated farming strategies to improve productivity and profitability [5,6]. At the same time, such biological divergence also suggests that males and females may respond differently to nutritional interventions, particularly those targeting redox balance, tissue quality, and metabolic regulation. Therefore, the development of precise and sex-specific dietary strategies is of particular importance for this species.

The use of natural antioxidants has emerged as an effective nutritional strategy to alleviate oxidative stress and improve the performance and product value of aquatic animals [7]. Among the carotenoids commonly used in aquafeeds, astaxanthin and β-carotene are two representative lipophilic pigments with important biological functions [8,9]. astaxanthin is widely recognized for its exceptional antioxidant activity and has been shown to enhance total antioxidant capacity, maintain glutathione homeostasis, promote pigmentation, and delay the deterioration of muscle quality in crustaceans [10]. β-carotene, as an important provitamin A precursor, also exhibits antioxidant properties, regulates lipid metabolism, and has a clear cost advantage compared with astaxanthin [11]. These characteristics suggest that astaxanthin and β-carotene may not only exert individual effects, but may also show complementary or interactive functions when used together in aquafeeds.

Despite the broad use of carotenoids in aquaculture, current research on crustacean antioxidant nutrition has focused predominantly on the phenotypic effects of individual compounds, whereas the combined use of astaxanthin and β-carotene remains insufficiently understood. In particular, it is still unclear whether these two carotenoids interact synergistically, independently, or competitively in terms of intestinal absorption, in vivo transformation, and antioxidant network regulation. As a result, the effects of their dietary ratios on systemic redox homeostasis remain poorly defined. Moreover, sexual dimorphism is often overlooked in existing studies, even though the distinct energy allocation strategies of males and females are likely to produce sex-specific differences in carotenoid assimilation, utilization, and physiological responses [6,12].

Another important limitation is that most previous studies have focused mainly on systemic physiological traits [13,14], with relatively limited attention to market-relevant product quality endpoints. In crustaceans, oxidation of proteins and lipids is closely linked to texture deterioration, pigment instability, and flavor loss. Therefore, improving antioxidant status may have consequences not only for health and growth, but also for pigmentation, muscle quality, and flavor profile. However, direct experimental evidence connecting carotenoid-based antioxidant nutrition with these edible quality traits in *M. rosenbergii* remains scarce. Thus, a systematic evaluation integrating antioxidant status, pigmentation, muscle quality, and flavor characteristics is still needed.

To address these gaps, the present study conducted a 77-day feeding trial to evaluate the effects of dietary astaxanthin and β-carotene combinations provided at seven graded ratios in male and female *M. rosenbergii*. The objectives were to determine whether combined carotenoid supplementation could improve antioxidant status, pigmentation, muscle quality, and flavor profile, to identify the optimal dietary combination, and to clarify whether these responses differed between sexes. The results are expected to provide a scientific basis for the development of precise, sex-differentiated functional aquafeeds for this commercially important species.

## 2. Materials and Methods

### 2.1. Ethics Statement

The handling and culture of the animals used in this research study were carried out in compliance with the guidelines established by the Animal Ethics Committee of Shanghai Ocean University (Shanghai, China), following the approved protocol numbers SHOU-DW-2025-172 (Approved on 10 April 2025).

### 2.2. Carotenoid Sources and Preparation of Experimental Diets

Seven iso-nitrogenous and iso-lipidic semi-purified pelleted diets were formulated to contain 47% crude protein and 9% crude lipid, as shown in Table 1. The control diet was prepared without carotenoid supplementation. The dietary carotenoid levels were selected based on previous carotenoid supplementation studies in crustaceans and practical feed formulation considerations. Mix1–Mix5 were formulated to contain a similar total carotenoid level of approximately 160 mg/kg but different astaxanthin/β-carotene ratios, allowing for comparison of ratio-dependent effects and evaluation of whether β-carotene could partially replace astaxanthin. Mix6, containing 160 mg/kg astaxanthin and 160 mg/kg β-carotene, was included as a high combined-dose treatment to determine whether simultaneous supplementation of both carotenoids produced additional benefits. In addition, the Control, Mix1, Mix5, and Mix6 groups constituted a 2 × 2 factorial design for evaluating the main and interaction effects of astaxanthin and β-carotene.

The carotenoid sources were as follows: astaxanthin from CAROPHYLL^®^ Pink (10% synthetic astaxanthin; DSM Nutrition, Heerlen, Switzerland) and β-carotene from POVIMIX^®^ β-Carotene (10% synthetic β-carotene; DSM Nutrition, Heerlen, Switzerland). Dietary protein was mainly supplied by Peruvian fish meal, soybean meal, meat meal, and crystalline amino acids, whereas fish oil, soybean oil, and lecithin served as the principal lipid sources.

For diet preparation, all dry ingredients were finely ground, passed through a sieve (<150 μm), and thoroughly mixed. A premixed blend of lipids and other lipid-soluble ingredients was then added and homogenized. Deionized water equivalent to 30% of the total dry ingredient weight was gradually added to form a uniform dough. The dough was extruded through a single-screw extruder to produce pellets with a diameter of 1.5 mm. The pellets were dried in a mechanical convection oven at 35 °C until the moisture content reached approximately 10%. Finally, the diets were sealed in plastic bags and stored at −20 °C until use.

### 2.3. Feeding Trial and Experimental Conditions

Postlarvae (PL25) used in this study were obtained from Zhejiang Zhongyi Aquatic Seed Co., Ltd. (Zhejiang, China). A total of 4000 postlarvae were first reared in a nursery phase for 2 months at the Binghai Aquaculture Base of Shanghai Ocean University under water temperatures of 18–23 °C and pH 7.8–8.5. Thereafter, 3360 juveniles of uniform size were selected and randomly allocated to seven indoor concrete tanks (5.0 m × 11.0 m × 1.2 m) for a 1-week acclimation period, during which all animals were fed the control diet. After acclimation, the juveniles had an average body length of 2.5 cm and an average body weight of 0.32 g.

According to Shen et al., *M. rosenbergii* juveniles with a body length of 2.5 cm can be reliably sexed [15]. Each concrete tank was assigned one experimental diet throughout the feeding trial, with all rearing and management conditions kept identical among tanks. Within each tank, six independent polyethylene net cages (1.0 m × 1.0 m × 1.2 m; mesh size, 0.5 cm) were suspended. Three cages were used for female prawns and the other three for male prawns. To minimize positional effects, the cages were arranged symmetrically along the two 5.0 m sides of each tank, with three cages evenly distributed on each side in an alternating sequence of male and female cages. In total, the experiment consisted of 42 independent rearing units.

The feeding trial lasted for 77 days. During this period, prawns were hand-fed four times daily at 06:30, 11:30, 17:30, and 21:30, with a daily ration corresponding to 5–8% of body weight. Individuals were randomly sampled weekly to determine body weight, and feeding rates were adjusted accordingly. Uneaten feed pellets and feces were removed daily by siphoning. All uneaten feed samples were stored at −20 °C and subsequently freeze-dried to constant weight for calculation of actual feed intake and feed conversion ratio (FCR).

Water quality parameters were monitored daily to maintain stable rearing conditions. Water temperature was maintained at 28 ± 3 °C, dissolved oxygen was kept above 6.0 mg/L, total ammonia nitrogen remained below 0.05 mg/L, pH ranged from 7.8 to 8.5, and salinity was maintained at 1–2‰.

### 2.4. Samples Collection

At the end of the 77-day feeding trial, all prawns were fasted for 12 h prior to final sampling [16]. The number of surviving prawns in each cage was recorded, and their final body weights were measured.

For whole-body composition and carotenoid analyses, prawns from all replicate cages within each treatment were pooled, and three individuals were randomly selected from the pooled sample. For hepatopancreatic analyses, 15 prawns from each replicate cage were randomly selected, dissected, and the hepatopancreas was collected into 2 mL centrifuge tubes. The samples were immediately snap-frozen in liquid nitrogen and stored at −80 °C until further analysis. In addition, three prawns from each replicate cage were randomly sampled for body pigmentation assessment.

### 2.5. Proximate Composition and Carotenoid Content

After freeze-drying and grinding, whole-body prawn and diet samples were analyzed for proximate composition according to standard AOAC procedures [17]. Crude protein (N × 6.25) was determined by the Dumas combustion method (AOAC method 990.03) using a CHN828 Dumas Nitrogen Analyzer (LECO, St. Joseph, MI, USA). Crude lipid was extracted according to Folch et al. and quantified gravimetrically after drying under a nitrogen stream [18]. Ash content was determined by pre-incineration on a hot plate followed by combustion in a muffle furnace (KSL-1200X, Hefei, China) at 550 °C for 6 h. Moisture content was measured by oven drying (DHG-9245A, Bluepard Instruments, Shanghai, China) at 105 °C for 5 h.

Carotenoid contents in whole-body and diet samples were determined using a Waters ACQUITY UPLC system (Waters Corporation, Milford, MA, USA) equipped with a Waters ACQUITY H-Class BEH C18 column (1.7 μm, 2.1 mm × 150 mm) [19]. Body pigmentation was further evaluated using a handheld colorimeter (Chroma Meter CR-400, Konica Minolta Sensing Inc., Osaka, Japan).

### 2.6. Activities of Hepatopancreatic Antioxidant Parameters and Digestive Enzymes

Hepatopancreas samples were processed according to the method of Xue et al. [20]. Briefly, hepatopancreatic tissue was homogenized in prawn physiological saline (NaCl 28.4 g/L, MgCl_2_∙6H_2_O 1 g/L, MgSO_4_∙7H_2_O 2 g/L, CaCl_2_∙2H_2_O 2.25 g/L, KCl 0.7 g/L, glucose 1 g/L, Hepes 2.38 g/L) at a ratio of 1: 5 (*w*/*v*) and then centrifuged at 8000 rpm for 15 min at 4 °C using a TG-16 centrifuge (Xiangyi, Hunan, China). The resulting supernatant was collected for the determination of antioxidant-related parameters, including total antioxidant capacity (T-AOC), the ratio of reduced to oxidized glutathione (GSH/GSSG), and malondialdehyde (MDA), as well as digestive enzyme activities, including trypsin, lipase, and amylase. All biochemical indices were measured using commercial assay kits supplied by Nanjing Jiancheng Bioengineering Institute (Nanjing, China) according to the manufacturer’s instructions. Absorbance was recorded using a Synergy Mx multimode microplate reader (BioTek Instruments, Winooski, VT, USA). Enzyme activities were expressed as specific activities. One unit of enzyme activity was defined according to the manufacturer’s instructions as the amount of enzyme required to catalyze the corresponding substrate reaction per unit time under the assay conditions. Specific activity was calculated by normalizing enzyme activity to soluble protein concentration and expressed as U/mg protein.

T-AOC was determined using the ABTS method (kit no. A084-1-1), which is based on the inhibition of ABTS^+^ radical formation by antioxidants present in the sample. The GSH/GSSG ratio was measured using a DTNB-based cycling method (kit no. A061-1-2). In this assay, total glutathione (T-GSH) and oxidized glutathione (GSSG) were first measured, and reduced glutathione (GSH) was subsequently calculated to obtain the GSH/GSSG ratio. MDA content was quantified using the thiobarbituric acid (TBA) method (kit no. A003-1-2), in which MDA reacts with TBA to form a red adduct measured at 532 nm.

Trypsin activity was determined colorimetrically (kit no. A080-2-2) based on the hydrolysis of the substrate ethyl arginine, which results in an increase in absorbance at 253 nm. Lipase activity was measured using a colorimetric assay (kit no. A054-1-1), in which triglyceride hydrolysis leads to micellar disintegration and a corresponding decrease in turbidity. Amylase activity was assayed colorimetrically (kit no. C016-1-1). In this method, α-amylase hydrolyzes starch into glucose, maltose, and dextrin; the residual starch then reacts with iodine to form a blue complex, and the extent of starch hydrolysis is determined from the intensity of the blue color.

### 2.7. Determination of Non-Volatile and Volatile Flavor Substances

Free amino acid content was determined with slight modifications to the method of Zhang et al. [21]. Briefly, 2.0 g of prawn muscle was accurately weighed and homogenized with 5 mL of 5% trichloroacetic acid (TCA) for 120 s. The homogenate was sonicated for 30 min and then allowed to stand at room temperature for 2 h. After centrifugation at 10,000 rpm for 15 min at 4 °C, the extraction was repeated twice. The resulting extracts were pooled, mixed with 2 mL of 3 mol/L NaOH, and diluted to a final volume of 25 mL with 5% TCA. After filtration through a 0.22 μm aqueous syringe filter, the filtrate was analyzed using an automatic amino acid analyzer (LA8080, Hitachi High-Tech Corp., Tokyo, Japan). Individual amino acids were identified and quantified by comparison with external amino acid standards, and the results were expressed as mg/100 g muscle.

Nucleotide content was quantified according to Zhang et al. [21], with minor modifications. Briefly, 3.0 g of prawn muscle was homogenized with 10 mL of 10% perchloric acid (PCA) for 60 s, sonicated for 5 min under chilled conditions, and centrifuged at 10,000 rpm for 15 min at 4 °C. The supernatant was collected, and the residue was extracted twice more with 5 mL of 5% PCA under the same conditions. All supernatants were combined, and the pH was adjusted to 5.80 ± 0.02 using 6 mol/L or 1 mol/L KOH. After standing at 4 °C for 30 min, the solution was adjusted to 50 mL, filtered through a 0.22 μm aqueous membrane, and analyzed by ultraviolet detection using a Waters e2695 high-performance liquid chromatography system (Waters Corporation, Milford, MA, USA) equipped with an Inertsil ODS-E C18 reverse-phase column (5 μm, 4.6 mm × 250 mm; GL Sciences Inc., Tokyo, Japan). Nucleotides were detected by ultraviolet detection, identified by comparison with authentic standards, and quantified using external standard curves.

The volatile flavor profile and fingerprint of prawn muscle were characterized using a FlavourSpec^®^ HS-GC-IMS instrument (G.A.S., Dortmund, Germany), following Zhang et al. with minor modifications [22]. Briefly, 0.5 g of sample was placed in a 20 mL headspace vial and incubated at 60 °C for 20 min with agitation at 500 rpm. Subsequently, 500 μL of headspace gas was automatically injected in splitless mode at 85 °C. Volatile compounds were separated on an MXT-WAX capillary column (30 m × 0.53 mm, 1 μm film thickness) maintained at 60 °C. High-purity nitrogen (≥99.999%) was used as the carrier gas under the following linear flow program: 2 mL/min for 2 min, held at 2 mL/min for 8 min, increased to 10 mL/min over 10 min and held for 10 min, and finally increased to 100 mL/min and maintained for 10 min. The IMS detector was operated at 45 °C, with high-purity nitrogen (≥99.999%) used as the drift gas at a constant flow rate of 150 mL/min.

### 2.8. Pigmentation and Muscle Texture Determination

Individual prawns were cooked in a boiling water bath for 3 min. Immediately after cooking, the samples were transferred to tap water at approximately 5 °C and cooled in the dark prior to color measurement. Color parameters were determined according to the method of Wang et al. using a CR-400 colorimeter (Konica Minolta Sensing Inc., Osaka, Japan) under standardized fluorescent lighting [23]. Based on the Commission on Illumination (CIE) color space system, three color parameters of the cooked prawn were recorded: L^*^ (lightness), a^*^ (redness), and b^*^ (yellowness) [24].

For texture analysis, excess surface moisture was removed from the cooked prawns using filter paper. After removal of the head and tail, the uniform middle portion of the muscle was trimmed into a standardized cylindrical shape and placed on the platform of a texture analyzer. Texture measurements were performed using a two-cycle compression texture profile analysis (TPA) mode. The testing conditions were set as follows: pre-test speed, 1.0 mm/s; test speed, 2.0 mm/s; post-test speed, 1.0 mm/s; trigger force, 5.0 g; compression strain, 30%; and interval time between the two compressions, 5 s. Hardness, springiness, cohesiveness, and chewiness were automatically calculated and recorded by the instrument.

### 2.9. Calculations, Statistical Analysis, and Visualization

The formulas used to calculate the growth parameters, which encompass weight gain (WG), specific growth rate (SGR), survival rate, and feed conversion ratio (FCR), are detailed as follows:

WG (%) = [(final weight-initial weight)/initial weight] × 100

SGR (%/day) = [(Ln final weight-Ln initial weight)/duration] × 100

Survival (%) = (final number of prawn/initial number of prawn) × 100

FCR = dry weight of feed consumed (g)/live weight gain (g)

For each dietary treatment, three independent net cages were used for each sex, and each cage was considered one biological replicate. Therefore, all data were analyzed using three biological replicates per sex per dietary treatment.

The Taste Active Value (TAV) was used to evaluate the contribution of individual taste compounds to the overall flavor of the prawns. A compound with a TAV > 1 was considered to contribute significantly to the overall flavor profile [25]. TAV was calculated using the following equation:

$$\mathrm{TAV}\;=\;\frac{C}{T}$$

In the equation, C represents the absolute concentration of the flavour compound (mg/100 g) and T denotes its sensory threshold (mg/100 g).

Equivalent umami concentration (EUC) was used to evaluate the synergistic umami effect between umami amino acids and 5′-flavor nucleotides. According to the method of Xue et al. [26], EUC was calculated as follows:

EUC=∑aibi+1218(∑aibi)(∑ajbj)

EUC is expressed as grams of monosodium glutamate (MSG) equivalents per 100 g of sample. In this equation, aia_iai represents the concentration of umami amino acids (g/100 g), and bib_ibi represents their corresponding relative umami coefficients (Glu = 1, Asp = 0.077). Likewise, aja_jaj represents the concentration of 5′-nucleotides (g/100 g), and bjb_jbj represents their corresponding relative umami coefficients (IMP = 1, GMP = 2.3, AMP = 0.18). The constant 1218 is the synergistic coefficient used to account for the interaction between umami amino acids and nucleotides.

All data are presented as mean ± standard error of the mean (SEM). Statistical analyses were performed using SPSS version 26.0 (IBM Corp., Armonk, NY, USA). Overall differences among the seven dietary treatment groups were analyzed by one-way analysis of variance (ANOVA), followed by Tukey’s multiple comparison test. In addition, to evaluate the main and interaction effects of astaxanthin and β-carotene, data from the Control, Mix1, Mix5, and Mix6 groups were further analyzed by two-way ANOVA in a 2 × 2 factorial design, with astaxanthin supplementation level (0 or 160 mg/kg) and β-carotene supplementation level (0 or 160 mg/kg) as fixed factors. These four groups were selected because they represented the full factorial combination of the presence or absence of astaxanthin and β-carotene at 160 mg/kg. Differences were considered statistically significant at *p* < 0.05.

### 2.10. Integrated CRITIC-TOPSIS Framework for Comprehensive Evaluation

To comprehensively compare dietary treatments, an integrated CRITIC-TOPSIS framework was established based on our previous evaluation strategy, with modifications to indicator selection, hierarchical structure, and weighting procedures to fit the present study [20]. The analysis incorporated multiple biologically relevant traits related to antioxidant status, growth, pigmentation, muscle quality, and flavor profile in male and female *M. rosenbergii*.

Briefly, candidate indicators were first screened according to biological relevance and practical interpretability. Each retained variable was then classified as either benefit-oriented (the larger, the better) or cost-oriented (the smaller, the better), and standardized before subsequent analysis. To reflect both relative dispersion and inter-indicator conflict, objective weights at the indicator level were calculated using a modified CRITIC method, in which the coefficient of variation was used in place of the standard deviation. For higher-level subclasses, composite weights were generated by combining expert-derived AHP weights with CRITIC-based objective weights.

The weighted standardized matrix was subsequently subjected to TOPSIS analysis to determine the relative closeness of each treatment to the ideal solution. Scores were first obtained at the indicator level and then hierarchically aggregated to generate subclass, class, and overall comprehensive evaluation scores. All computations were performed using Python 3.10 and R 4.4.2.

## 3. Results

### 3.1. Growth Performance and Whole-Body Proximate Composition of M. rosenbergii

**Figure 1:**
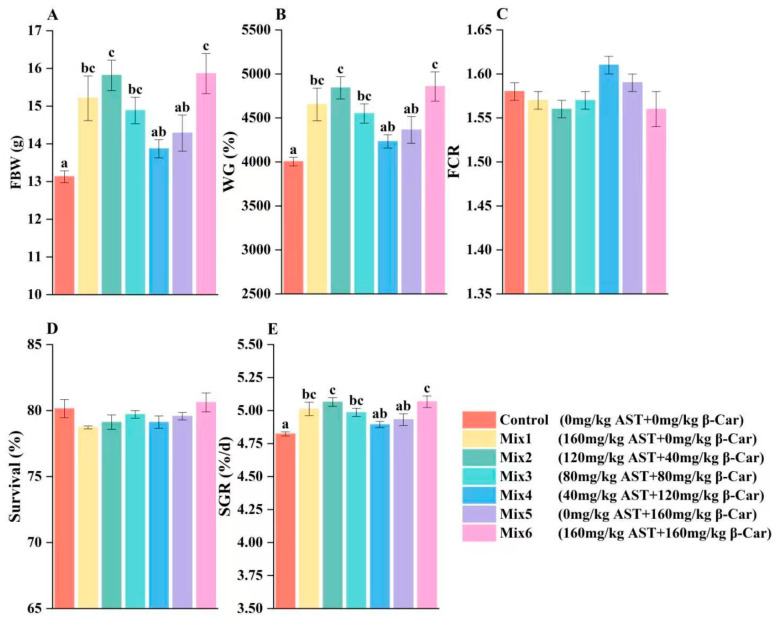
Growth performance and survival rate of male *M. rosenbergii* fed the experimental diets for 77 days. Values are expressed as means ± SEM (*n* = 3 biological replicates). Different lowercase letters above the bars indicate significant differences among treatments (*p* < 0.05). (**A**) final body weight (FBW); (**B**) weight gain (WG); (**C**) feed conversion ratio (FCR); (**D**) survival rate; (**E**) specific growth rate (SGR).

**Figure 2:**
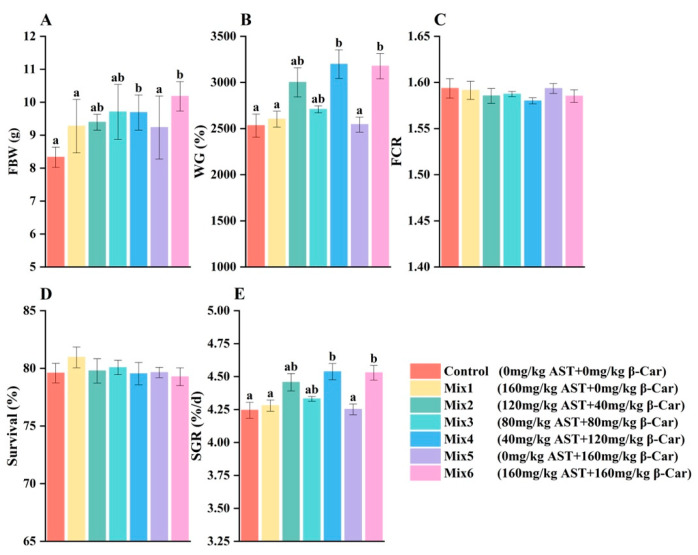
Growth performance and survival rate of female *M. rosenbergii* fed the experimental diets for 77 days. Values are expressed as means ± SEM (*n* = 3 biological replicates). Different lowercase letters above the bars indicate significant differences among treatments (*p* < 0.05). (**A**) final body weight (FBW); (**B**) weight gain (WG); (**C**) feed conversion ratio (FCR); (**D**) survival rate; (**E**) specific growth rate (SGR).

At the end of the 77-day feeding trial, show in Figure 1, survival rate and feed conversion ratio (FCR) of male *M. rosenbergii* did not differ significantly among the dietary treatments (*p* > 0.05). In contrast, growth performance was significantly affected by carotenoid supplementation. Male prawns in the Mix2 and Mix6 groups showed significantly higher final body weight (FBW), weight gain (WG), and specific growth rate (SGR) than those in the control group (*p* < 0.01). The highest WG in males was observed in the Mix6 group, reaching 4857.29%. Similarly, in female prawns (Figure 2), survival rate and FCR remained stable across all treatment groups (*p* > 0.05). However, FBW, WG, and SGR were significantly higher in the Mix4 and Mix6 groups than in the control group (*p* < 0.01). The highest WG in females was recorded in the Mix4 group (3198.96%), followed closely by the Mix6 group (3177.08%).

Whole-body proximate composition was not significantly affected by dietary treatments in either sex (*p* > 0.05). As summarized in Table 2 and Table 3, astaxanthin exerted a highly significant main effect on male FBW, WG, and SGR (*p* < 0.01), whereas neither the main effect of β-carotene nor the interaction term was significant. In females, both astaxanthin and β-carotene had significant main effects on WG and SGR (*p* < 0.05), and significant interaction effects were also detected for these parameters.

### 3.2. Targeted Antioxidant Status

**Figure 3:**
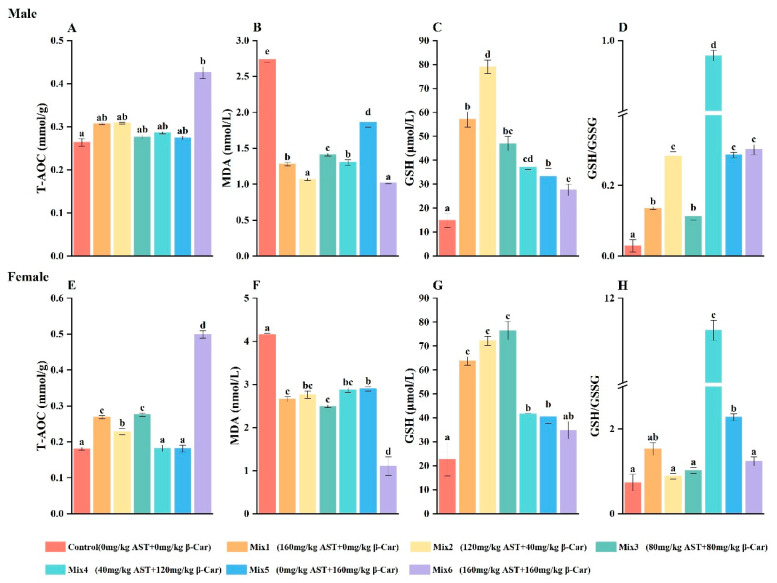
Effects of different dietary ratios of astaxanthin and β-carotene on antioxidant parameters in *M. rosenbergii* after 77 days of feeding. Values are expressed as means ± SEM (n = 3 biological replicates). Different lowercase letters above the bars indicate significant differences among treatments (*p* < 0.05). (**A**) total antioxidant capacity (T-AOC) of male prawns; (**B**) malondialdehyde (MDA) of male prawns; (**C**) reduced glutathione (GSH) of male prawns; (**D**) the ratio of reduced to oxidized glutathione (GSH/GSSG) of male prawns; (**E**) total antioxidant capacity (T-AOC) of female prawns; (**F**) malondialdehyde (MDA) of female prawns; (**G**) reduced glutathione (GSH) of female prawns; (**H**) the ratio of reduced to oxidized glutathione (GSH/GSSG) of female prawns.

Dietary supplementation with different carotenoid ratios significantly modulated the antioxidant status of *M. rosenbergii*. In male prawns (Figure 3A–D), the Mix6 group exhibited significantly higher total antioxidant capacity (T-AOC) and lower malondialdehyde (MDA) content than the control group (*p* < 0.01). A similar pattern was observed in females (Figure 3E–H), in which Mix6 also showed the highest T-AOC and the lowest MDA level (*p* < 0.01).

According to the two-way ANOVA summary in Table 2 and Table 3, both astaxanthin and β-carotene exerted highly significant main effects on T-AOC and MDA in males, and their interaction was also highly significant (*p* < 0.01). In females, astaxanthin, β-carotene, and their interaction all significantly affected T-AOC.

### 3.3. Digestive Enzyme Activities

**Figure 4:**
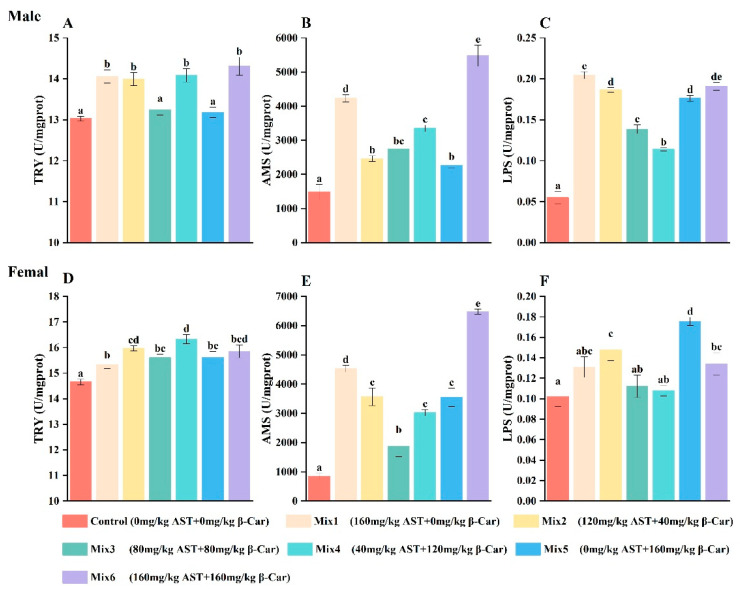
Effects of different ratios of astaxanthin and β-carotene on the digestive enzymes of *M. rosenbergii* after 77 days of cultivation. Values are expressed as means ± SEM (n = 3 biological replicates). Different lowercase letters above the bars indicate significant differences among treatments (*p* < 0.05). (**A**) trypsin (TRY) of male prawns; (**B**) amylase (AMS) of male prawns; (**C**) lipase (LPS) of male prawns; (**D**) trypsin (TRY) of female prawns; (**E**) tamylase (AMS) of female prawns; (**F**) lipase (LPS) of female prawns.

Dietary carotenoid supplementation also significantly affected digestive enzyme activities in *M. rosenbergii*. In male prawns (Figure 4A–C), trypsin (TRY), amylase (AMS), and lipase (LPS) activities differed significantly among treatments, with the highest AMS activity recorded in the Mix6 group (5479.54 U/mg protein). In females (Figure 4D–F), carotenoid-supplemented groups generally showed higher digestive enzyme activities than the control group (*p* < 0.05 or *p* < 0.01).

As summarized in Table 2 and Table 3, astaxanthin exerted highly significant main effects on AMS and LPS in males, and the interaction between astaxanthin and β-carotene was also highly significant for AMS activity (*p* < 0.01). In females, β-carotene and the interaction term showed highly significant effects on AMS activity.

### 3.4. Body Coloration and Total Carotenoid Deposition

**Figure 5:**
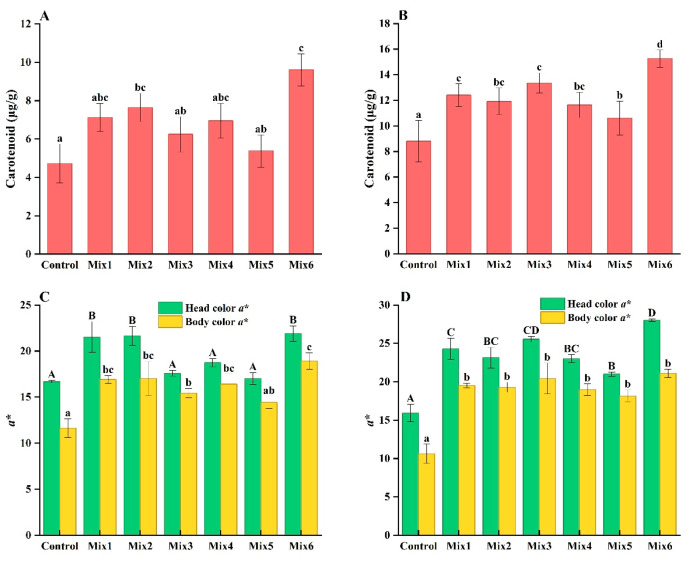
Effects of different ratios of astaxanthin and β-carotene on carotenoid deposition and body redness in *M. rosenbergii* after 77 days of feeding. Values are expressed as means ± SEM (n = 3 biological replicates). Different lowercase letters above the bars indicate significant differences among treatments (*p* < 0.05), while different uppercase letters indicate highly significant differences among treatments (*p* < 0.01). (**A**): Carotenoid content in male prawns; (**B**): Carotenoid content in female prawns; (**C**): body redness (a^*^) in male prawns; (**D**): body redness (a^*^) in female prawns.

Dietary carotenoid supplementation significantly enhanced pigment deposition in both sexes of *M. rosenbergii*. In males (Figure 5A,C), total muscle carotenoid content and head and body redness (a^*^) were significantly higher in the Mix6 group than in the control group (*p* < 0.05 or *p* < 0.01). In females (Figure 5B,D), all mixed-carotenoid treatments resulted in significantly higher total carotenoid contents and a^*^ values than the control, with the most pronounced response observed in Mix6.

As summarized in Table 2 and Table 3, astaxanthin exerted a highly significant main effect on total carotenoid content and body redness in males, and significant interaction effects were detected for head and body a^*^ values. In females, both astaxanthin and β-carotene significantly affected carotenoid deposition and redness, and their interaction had a highly significant effect on both head and body a^*^ values.

### 3.5. Muscle Texture Profile Analysis (TPA)

Texture profile analysis showed that dietary carotenoid supplementation significantly affected the muscle textural properties of *M. rosenbergii*. In male prawns, as shown in Table 4, muscle hardness and chewiness were significantly higher in the Mix2, Mix5, and Mix6 groups than in the control group (*p* < 0.05). In addition, significant differences in springiness and cohesiveness were observed among the male treatment groups (*p* < 0.05), with the highest springiness recorded in the Mix5 group. Female prawns showed a similar response, as shown in Table 5, although the pattern differed among treatments. Specifically, muscle hardness and chewiness were significantly higher in the Mix3 group than in the control group (*p* < 0.05). Significant intergroup differences in springiness and cohesiveness were also detected among the female treatments (*p* < 0.05).

### 3.6. Muscle Free Amino Acid Composition and Taste Activity Value (TAV)

Free amino acid (FAA) concentrations in male prawn (Table 6) muscle varied significantly among the dietary treatments. Marked differences were observed in several major taste-related amino acids, including glutamic acid (Glu), glycine (Gly), alanine (Ala), and arginine (Arg) (*p* < 0.01). In females (Table 7), Glu, Gly, and Ala also differed significantly among treatments, with the highest Glu content recorded in Mix6 (*p* < 0.01). As shown in Table 8 and Table 9, Taste activity value (TAV) analysis further showed that Gly, Ala, His, and Arg were the principal taste-active amino acids in both sexes, with values greater than 1.0 across all treatments.

As summarized in Table 2 and Table 3, astaxanthin exerted highly significant main effects on most FAAs in males, whereas significant interaction effects between astaxanthin and β-carotene were detected for several amino acids. In females, astaxanthin, β-carotene, and their interaction significantly affected the concentrations of Glu, Gly, Ala, Cys, Met, Lys, and His.

### 3.7. Muscle Flavor Nucleotides and Equivalent Umami Concentration (EUC)

**Figure 6:**
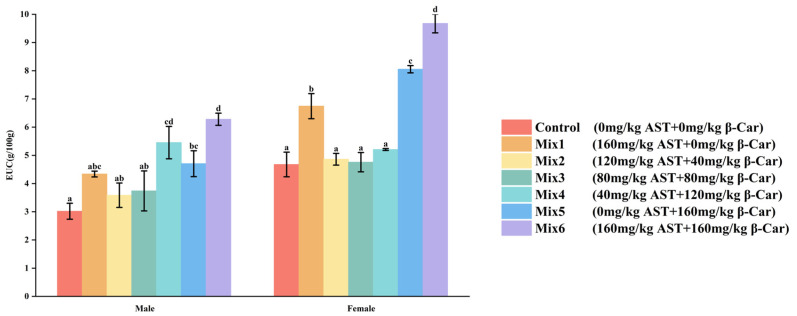
Effects of different ratios of astaxanthin and β-carotene on the antioxidant enzymes of *M. rosenbergii* after 77 days of feeding. Values are expressed as means ± SEM (n = 3 biological replicates). Different lowercase letters above the bars indicate significant differences among treatments (*p* < 0.05).

Taste-active nucleotide contents, as shown in Figure 6, including AMP, IMP, and GMP, differed significantly among treatments in male prawn muscle (*p* < 0.05 or *p* < 0.01). The Mix6 group showed a significantly higher EUC value (6.28 g/100 g) than the control group (*p* < 0.01). Female prawns also exhibited significant alterations in muscle nucleotide profiles, and EUC values were significantly elevated in Mix5 and Mix6, with the highest value recorded in Mix6 (9.68 g/100 g; *p* < 0.01).

According to Table 2 and Table 3, astaxanthin exerted significant main effects on all measured nucleotide components and EUC in males, whereas significant interaction effects were detected for ATP, GMP, and ADP. In females, both astaxanthin and β-carotene had highly significant main effects on most nucleotide components and EUC, and significant interaction effects were observed for ADP, HX, and AMP. However, no significant interaction between the two carotenoids was detected for EUC in either sex.

### 3.8. GC-IMS Fingerprint Profiles of Muscle Volatile Flavor Compounds

**Figure 7:**
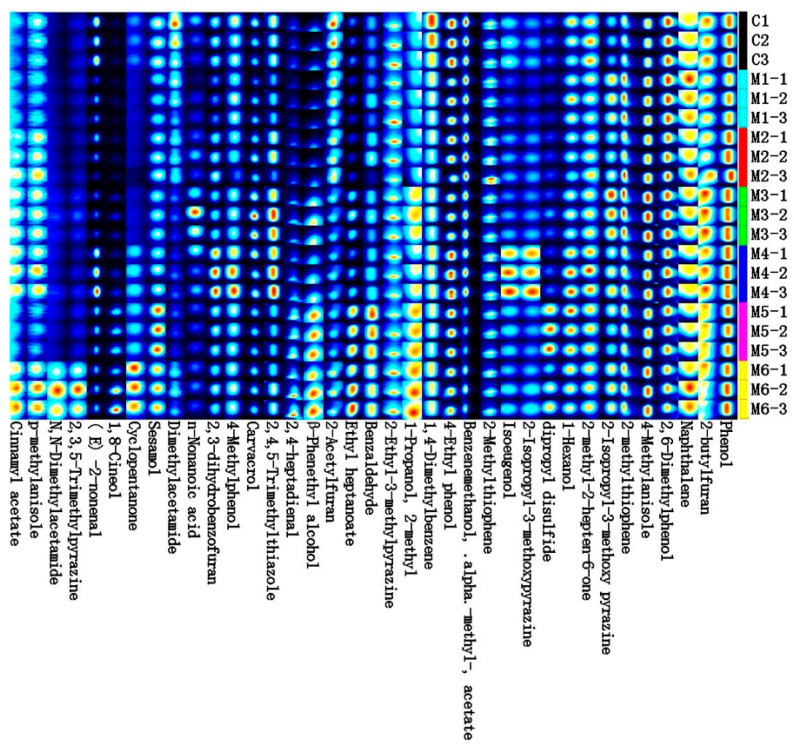
GC-IMS fingerprint chromatogram of male *M. rosenbergii*.

**Figure 8:**
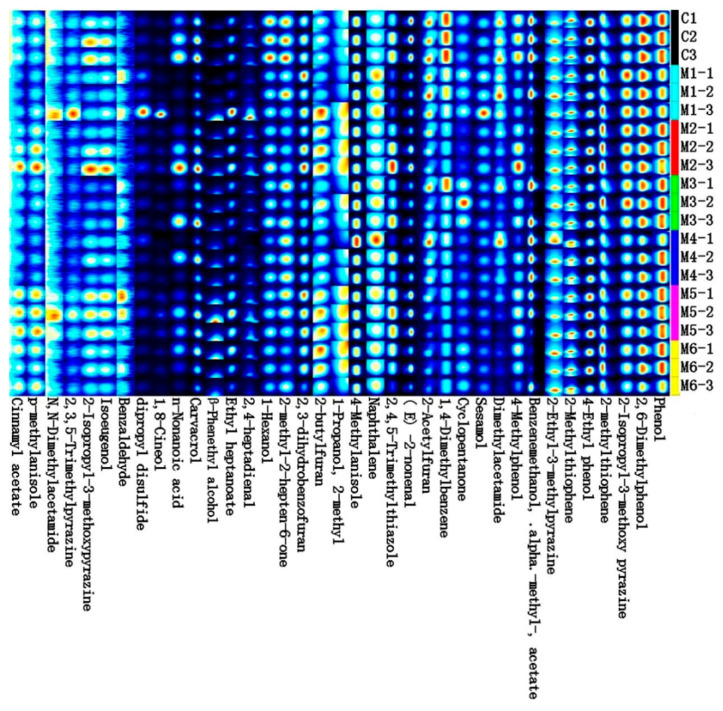
GC-IMS fingerprint chromatogram of female *M. rosenbergii*.

As shown in Figure 7 and Figure 8, two-dimensional gas chromatography–ion mobility spectrometry (GC-IMS) fingerprints revealed clear treatment- and sex-dependent differences in the volatile profiles of *M. rosenbergii* muscle. The relative abundance of several characteristic volatile organic compounds (VOCs) varied among dietary treatments, indicating that different ratios of astaxanthin and β-carotene affected volatile flavor composition in both sexes.

In male prawns, muscle VOCs showed pronounced treatment-specific variation. Although several baseline volatiles, including some phenol- and furan-related compounds, remained relatively stable across groups, marked differences were observed in the signal intensities of specific compounds. In particular, the Mix4 group showed distinct enrichment of isoeugenol and 1-hexanol, whereas the Mix6 group exhibited markedly enhanced signals for cinnamyl acetate and *p*-methylanisole. These results indicate that volatile flavor formation in male muscle was strongly influenced by dietary carotenoid ratio.

Female prawns showed a different response pattern. Compared with males, several carotenoid combinations in females induced a more coordinated increase in characteristic VOC signals. In particular, the Mix2, Mix5, and Mix6 groups consistently showed stronger signals for compounds such as benzaldehyde, 2,3,5-trimethylpyrazine, and 2,4-heptadienal. These results suggest clear sex-related differences in the volatile response of prawn muscle to dietary carotenoid supplementation.

### 3.9. Comprehensive Evaluation Analysis

**Figure 9:**
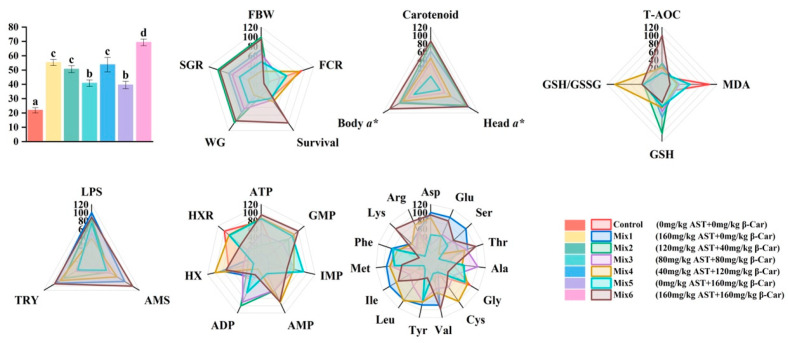
Comprehensive evaluation of male *M. rosenbergii* fed graded levels of astaxanthin and β-carotene. Different lowercase letters above the bars indicate significant differences among treatments (*p* < 0.05).

**Figure 10:**
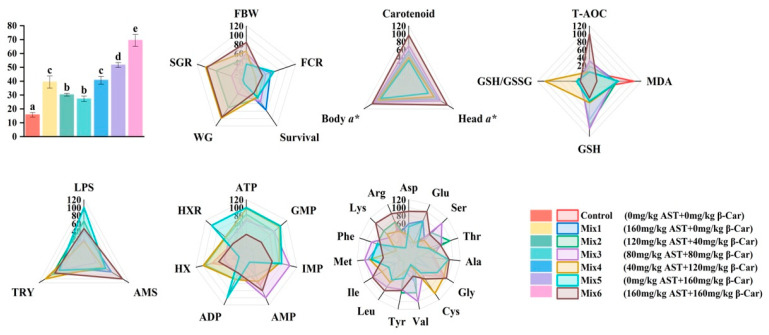
Comprehensive evaluation of female *M. rosenbergii* fed graded levels of astaxanthin and β-carotene. Different lowercase letters above the bars indicate significant differences among treatments (*p* < 0.05).

Comprehensive evaluation showed that different dietary ratios of astaxanthin and β-carotene produced distinct overall response patterns in male and female *M. rosenbergii* (Figure 9 and Figure 10). In males, the Mix6 treatment (160 mg/kg astaxanthin + 160 mg/kg β-carotene) achieved the highest comprehensive score, indicating the most favorable overall performance among all treatments. The Mix1 and Mix4 groups also ranked relatively high, whereas the β-carotene-only treatment (Mix5) showed a comparatively lower score among the carotenoid-supplemented groups.

A similar overall trend was observed in females, with the Mix6 group again achieving the highest comprehensive score. In addition, the Mix5 group showed a substantially higher comprehensive score than the astaxanthin-only group (Mix1), indicating a different response pattern between sexes under single-factor supplementation. Overall, the comprehensive evaluation results demonstrated that combined supplementation with astaxanthin and β-carotene, particularly at the high-dose level, produced the best overall performance in both male and female prawns.

### 3.10. Correlation Analysis Among Diverse Indicators

**Figure 11:**
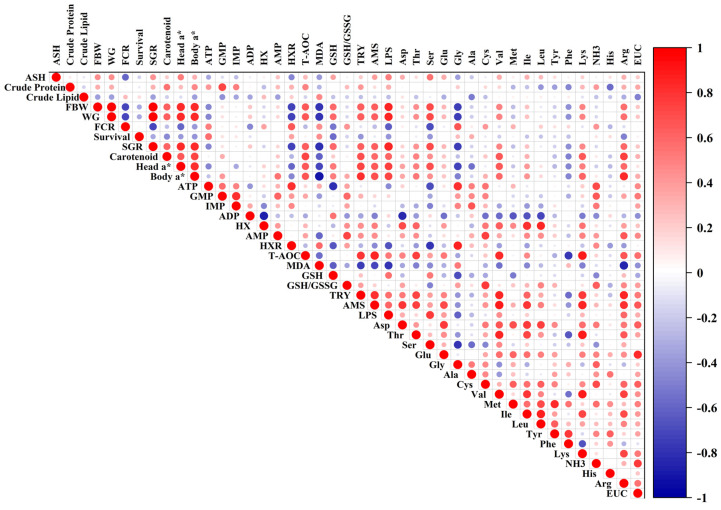
Correlation heatmap of multiple indicators in male *M. rosenbergii*. a* denotes redness value in CIELAB colour system.

As shown in Figure 11, extensive correlations were observed among the physiological and biochemical indicators in male *M. rosenbergii*. In particular, total antioxidant capacity (T-AOC), glutathione (GSH) content, and the GSH/GSSG ratio were significantly and positively correlated with growth-related traits, including final body weight (FBW), weight gain (WG), and specific growth rate (SGR), as well as with carotenoid content and coloration indices (head a^*^ and body a^*^). In contrast, malondialdehyde (MDA) showed strong negative correlations with these growth and pigmentation parameters, but a positive correlation with feed conversion ratio (FCR). In addition, most amino acids, such as Asp and Thr, were positively correlated with T-AOC and negatively correlated, to varying extents, with MDA.

**Figure 12:**
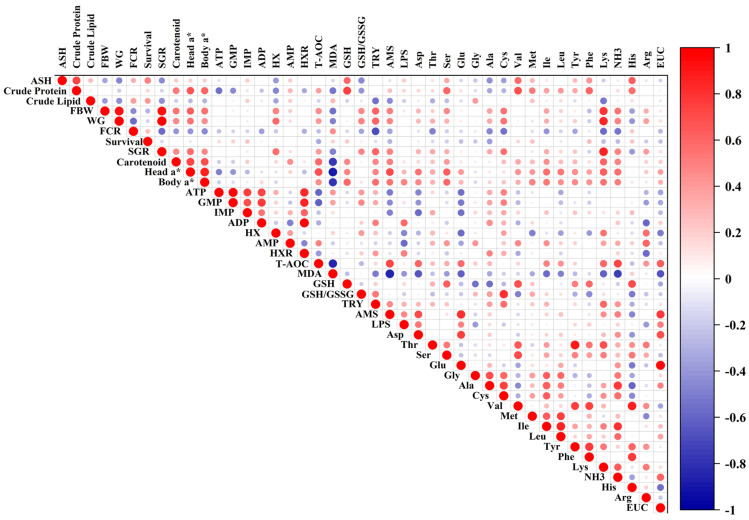
Correlation heatmap of multiple indicators in female *M. rosenbergii*. a* denotes redness value in CIELAB colour system.

A broadly similar correlation pattern was observed in female *M. rosenbergii* (Figure 12). T-AOC, GSH content, and the GSH/GSSG ratio were also significantly and positively associated with growth performance (FBW, WG, and SGR) and body coloration indices in females. Meanwhile, MDA showed strong negative correlations with growth and pigmentation traits and a positive correlation with FCR. Moreover, MDA was significantly and negatively correlated with several amino acids, particularly lysine (Lys), histidine (His), and arginine (Arg).

## 4. Discussion

### 4.1. Overview Effects of Combined Dietary Astaxanthin and β-Carotene

Maintaining redox homeostasis is a fundamental prerequisite for crustaceans to cope with environmental stress, preserve physiological stability, and maintain meat quality [27,28]. In the present study, dietary supplementation with astaxanthin (AST) and β-carotene, particularly at the high combined dose (Mix6), consistently improved antioxidant capacity, pigmentation, muscle quality, and flavor-related traits in *M. rosenbergii*. These improvements were accompanied by better growth performance and higher comprehensive evaluation scores in both sexes. Overall, the results indicate that combined carotenoid supplementation is more effective than single-factor supplementation in improving both physiological status and edible quality, although the magnitude and pattern of response differed between males and females.

### 4.2. Synergistic Regulation of Antioxidant Status by Astaxanthin and β-Carotene

The attenuation of lipid peroxidation is one of the most direct indicators of antioxidant efficacy. In the present study, prawns in the Mix6 group exhibited the highest total antioxidant capacity (T-AOC) together with the lowest malondialdehyde (MDA) levels, and two-way ANOVA further confirmed a significant interaction between astaxanthin and β-carotene on antioxidant-related traits. This synergistic response is biologically plausible because the two carotenoids differ in molecular polarity and membrane localization. Due to the polar keto and hydroxyl groups at both ends of its molecule, astaxanthin can span the phospholipid bilayer and provide transmembrane antioxidant protection. In contrast, β-carotene, as a strictly non-polar molecule, is more likely to accumulate within the hydrophobic core of the lipid bilayer [29]. Their combined presence may therefore reinforce oxidative protection across different membrane domains, forming a more effective reactive oxygen species (ROS)-scavenging barrier than either carotenoid alone [30,31,32].

### 4.3. Antioxidant Protection, Digestive Physiology, and Growth Performance

Improved antioxidant status was closely associated with better digestive function and growth. In crustaceans, the hepatopancreas is a central organ for digestion, lipid metabolism, immune defense, and detoxification, and is therefore highly vulnerable to oxidative stress [33]. In the present study, dietary carotenoid supplementation significantly increased the activities of major digestive enzymes, especially amylase, indicating that reduced oxidative injury helped preserve hepatopancreatic integrity and secretory capacity. Enhanced digestive enzyme activities would be expected to improve nutrient breakdown and assimilation efficiency [34,35], thereby contributing to the higher final body weight (FBW), weight gain (WG), and specific growth rate (SGR) observed in the Mix4 and Mix6 groups. Thus, the growth-promoting effect of carotenoid combinations appears to be mediated not only by direct antioxidant protection but also by improved digestive physiology and metabolic efficiency.

### 4.4. Carotenoid Deposition and Pigmentation Enhancement

Body coloration is an important indicator of both physiological status and commercial value in crustaceans. In the present study, combined carotenoid supplementation significantly increased both total muscle carotenoid content and redness (a^*^), with clear interaction effects between astaxanthin and β-carotene. This pattern is consistent with the “antioxidant resource allocation” concept, whereby reduced oxidative stress lowers the need to consume carotenoid molecules for radical scavenging, thereby allowing more pigments to be retained and deposited in tissues [36]. In addition, the significant interaction effects of astaxanthin and β-carotene on pigmentation suggest that these carotenoids may act cooperatively during intestinal absorption, transport, or tissue deposition, possibly through shared or mutually facilitative uptake and hemolymph lipoprotein transport pathways [37]. Therefore, the improvement in pigmentation likely reflects both direct carotenoid deposition and the indirect pigment-sparing effect of reduced oxidative stress.

### 4.5. Muscle Quality and Flavor Profile Improvement

The benefits of carotenoid supplementation extended beyond antioxidant status to edible quality traits. In terms of physical muscle properties, improved antioxidant protection likely stabilized cell membranes and reduced oxidative damage to structural proteins such as myofibrillar proteins, thereby contributing to the superior texture profiles observed in carotenoid-supplemented groups, especially Mix5 and Mix6 [38].

These effects also extended to flavor formation. Free amino acids (e.g., Glu and Gly) and flavor nucleotides (e.g., IMP and AMP), which are key contributors to umami and sweetness in aquatic products, are highly susceptible to degradation or loss under oxidative stress and tissue injury [38,39]. In the present study, the stronger antioxidant protection conferred by combined carotenoid supplementation appeared to stabilize these flavor precursors, resulting in markedly increased equivalent umami concentration (EUC), which reached 9.68 g/100 g in female prawns from the Mix6 group. At the same time, GC-IMS analysis revealed clear shifts in volatile organic compounds (VOCs), indicating that carotenoid supplementation not only preserved non-volatile taste-active compounds but also altered volatile flavor metabolism. Such changes suggest that targeted antioxidation may suppress the formation of off-flavors associated with excessive lipid oxidation, while favoring the accumulation of more desirable aromatic compounds, including esters and aromatics [40]. Together, these findings indicate that carotenoid-mediated antioxidant protection can simultaneously improve muscle texture, flavor precursor retention, and volatile flavor profile in *M. rosenbergii*.

### 4.6. Sex-Specific Responses to Combined Carotenoid Supplementation

A notable feature of the present study was the clear sexual dimorphism in response to carotenoid nutrition. Although Mix6 produced the best overall performance in both sexes, males and females differed in their responses to single-factor supplementation and in the pattern of volatile flavor remodeling. Males appeared to respond more strongly to direct astaxanthin supplementation, whereas females showed relatively greater responsiveness to β-carotene under single-factor conditions. This sex-specific pattern may be related to differences in energy allocation and lipid metabolism between male and female prawns. During ovarian development, females require substantial lipid mobilization, oxidation, and transport, which likely increases their endogenous antioxidant demand and alters their handling of carotenoids and flavor precursors [41,42]. As a result, exogenous composite antioxidants may exert a more systemic influence on the whole-body lipid metabolic network and volatile flavor metabolism in females. In contrast, males allocate more resources to somatic growth and thus may benefit more directly from astaxanthin-associated effects on antioxidant defense, pigmentation, and growth.

### 4.7. Practical Implications and Study Limitations

From a practical perspective, the present results support the use of combined astaxanthin and β-carotene supplementation as a functional feeding strategy for giant freshwater prawns. In particular, the superior performance of Mix6 indicates that combined carotenoid nutrition may be more effective than single-carotenoid supplementation in simultaneously improving antioxidant status, body coloration, muscle quality, and flavor-related traits. The sex-dependent response patterns further imply that future feed formulation may benefit from more precise, sex-oriented carotenoid nutrition strategies.

Several limitations should also be acknowledged. First, the present study provides physiological and biochemical evidence for synergistic carotenoid action, but the underlying molecular mechanisms were not directly verified. Second, GC-IMS analysis reveals differences in volatile fingerprints, but does not replace sensory evaluation. Third, the present conclusions were obtained under a single feeding duration and defined dietary inclusion levels, and therefore their generality under other farming conditions remains to be tested. Future studies should combine transcriptomic or enzymatic pathway analyses with sensory validation to clarify how astaxanthin and β-carotene jointly regulate antioxidant metabolism, carotenoid deposition, and flavor formation in male and female *M. rosenbergii*.

## 5. Conclusions

Dietary combinations of astaxanthin and β-carotene effectively enhanced antioxidant status, pigmentation, muscle quality, and flavor profile in *M. rosenbergii*. The Mix6 treatment showed the best overall performance in both males and females, indicating that combined high-dose supplementation was the most effective strategy. Clear sex-dependent responses were observed, with males responding more strongly to astaxanthin and females showing greater responsiveness to β-carotene under single-factor supplementation. These results suggest that combined carotenoid supplementation is a promising strategy for improving both physiological performance and edible quality in giant freshwater prawns. Future studies should combine response surface methodology-based optimization, longer-term culture trials, transcriptomic and metabolomic analyses, and intestinal absorption studies to further determine the optimal dietary ratio and inclusion level of astaxanthin and β-carotene and to clarify the mechanisms underlying sex-specific carotenoid utilization in *M. rosenbergii*.

## Figures and Tables

**Table 1:** Formulation and biochemical composition of the experimental diets (g/kg, dry matter basis).

Parameters	Treatments						
	Control	Mix1	Mix2	Mix3	Mix4	Mix5	Mix6
Peruvian fish meal ^a^	30.00	30.00	30.00	30.00	30.00	30.00	30.00
Meat meal ^a^	12.00	12.00	12.00	12.00	12.00	12.00	12.00
Soybean meal ^a^	22.00	22.00	22.00	22.00	22.00	22.00	22.00
Spray dried blood powder ^a^	3.00	3.00	3.00	3.00	3.00	3.00	3.00
Beer yeast ^a^	3.00	3.00	3.00	3.00	3.00	3.00	3.00
Squid paste ^a^	2.00	2.00	2.00	2.00	2.00	2.00	2.00
Fish oil ^a^	2.00	2.00	2.00	2.00	2.00	2.00	2.00
Soybean lecithin ^a^	1.00	1.00	1.00	1.00	1.00	1.00	1.00
Cellulose ^a^	2.50	2.34	2.34	2.34	2.34	2.34	2.18
Wheat flour ^a^	10.00	10.00	10.00	10.00	10.00	10.00	10.00
α-starch ^a^	6.00	6.00	6.00	6.00	6.00	6.00	6.00
Mineral mixture ^b^	1.50	1.50	1.50	1.50	1.50	1.50	1.50
Vitamin mixture ^c^	1.00	1.00	1.00	1.00	1.00	1.00	1.00
Ca(H_2_PO_4_)_2_ ^a^	2.00	2.00	2.00	2.00	2.00	2.00	2.00
Choline chloride ^a^	0.50	0.50	0.50	0.50	0.50	0.50	0.50
Vitamin C ^a^	0.30	0.30	0.30	0.30	0.30	0.30	0.30
Methionine ^d^	0.20	0.20	0.20	0.20	0.20	0.20	0.20
Glutamine ^d^	1.00	1.00	1.00	1.00	1.00	1.00	1.00
Astaxanthin ^e^	0	0.16	0.12	0.08	0.04	0	0.16
β-Carotene ^f^	0	0	0.04	0.08	0.12	0.16	0.16
Total	100.00	100.00	100.00	100.00	100.00	100.00	100.00
Proximate composition (dry weight basis, %)							
Crude protein	44.98	45.17	45.09	44.66	44.60	44.75	44.88
Crude lipid	8.66	8.97	8.30	8.34	8.87	8.06	8.39
Ash	10.34	10.63	10.58	10.25	11.16	10.37	10.64
Total carotenoids (mg/kg)	ND ^g^	159.87	161.18	158.17	165.06	157.47	323.02

^a^ Yuehai Feed Group Co., Ltd., Zhejiang, China. ^b^ Mineral mixture (15 g/kg diet): C_6_H_10_CaO_6_·5H_2_O, 0.76 g; K_2_HPO_4_, 5.23 g; MgSO_4_.7H_2_O, 5.36 g; NaH_2_PO_4_, 3.07 g; C_6_H_5_O_7_Fe·5H_2_O, 0.10 g; CuSO_4_.5H_2_O, 0.15 g; ZnSO_4_·7H_2_O, 0.21 g; CoCl_2_, 0.03 g; MnSO_4_·H_2_O, 0.09 g. ^c^ Vitamin mixture (10 g/kg diet) (vitamin A free): *p*-aminobenzoic acid 0.0767 g; biotin 0.0033 g; inositol 3.0567 g; nicotinic acid 0.3067 g, Ca-pantothenate 0.4600 g; pyridoxine-HCl 0.0933 g; riboflavin 0.0600 g; thiamine-HCl 0.0300 g; menadione 0.0300 g, α-tocopherol 0.1533 g; cyanocobalamin 0.00067 g; cholecalciferol 0.0100 g; stay-C 1.1333 g, folic acid 0.00667 g; choline chloride 4.5833 g. ^d^ Shanghai Macklin Biochemical Co., Ltd., Shanghai, China. ^e^ Astaxanthin (100 mg/kg): CAROPHYLL^®^ Pink containing 10% Synthetic astaxanthin made by DSM Nutrition, Heerlen, Switzerland. ^f^ β-carotene (300 mg/kg): POVIMIX^®^ β-Carotene containing 10% Synthetic β-carotene made by DSM Nutrition, Heerlen, Switzerland. ^g^ ND: not detected.

**Table 2:** Two-way ANOVA results for the effects of astaxanthin, β-carotene, and their interaction on multiple indices in male *M. rosenbergii*.

Parameters	*p* Values		
	Astaxanthin	β-Carotene	Interaction
Body weight	0.01	0.09	0.61
WG	0.01	0.09	0.61
SGR	0.01	0.08	0.54
Carotenoid	0.01	0.30	0.10
Head color a^*^	0.01	0.04	0.02
Body color a^*^	0.01	0.88	0.02
ATP	0.01	0.01	0.01
GMP	0.11	0.01	0.03
IMP	0.02	0.01	0.32
ADP	0.01	0.01	0.01
HX	0.01	0.01	0.01
AMP	0.02	0.04	0.32
HXR	0.01	0.03	0.01
T-AOC	0.01	0.01	0.01
MDA	0.01	0.01	0.01
GSH	0.35	0.77	0.23
GSH/GSSG	0.60	0.09	0.69
LPS	0.01	0.23	0.77
TRY	0.15	0.60	0.90
AMS	0.01	0.01	0.01
Asp	0.01	0.01	0.01
Thr	0.01	0.93	0.01
Ser	0.07	0.21	0.01
Glu	0.01	0.11	0.22
Gly	0.01	0.10	0.14
Ala	0.01	0.01	0.26
Cys	0.04	0.04	0.13
Val	0.01	0.55	0.25
Met	0.28	0.57	0.03
Ile	0.01	0.04	0.83
Leu	0.01	0.01	0.01
Tyr	0.08	0.95	0.01
Phe	0.50	0.24	0.01
Lys	0.01	0.01	0.01
His	0.14	0.96	0.01
Arg	0.01	0.03	0.89
EUC	0.01	0.01	0.68

Note: A *p*-value < 0.05 for astaxanthin or β-carotene indicates a significant main effect, whereas a *p*-value < 0.05 for the interaction term indicates a significant interaction between astaxanthin and β-carotene.

**Table 3:** Two-way ANOVA results for the effects of astaxanthin, β-carotene, and their interaction on multiple indices in female *M. rosenbergii*.

Parameters	*p* Values		
	Astaxanthin	β-carotene	Interaction
Body weight	0.69	0.67	0.38
WG	0.01	0.03	0.03
SGR	0.02	0.04	0.04
Carotenoid	0.01	0.03	0.45
Head color a^*^	0.01	0.01	0.01
Body color a^*^	0.01	0.01	0.01
ATP	0.01	0.01	0.19
GMP	0.01	0.01	0.82
IMP	0.01	0.01	0.25
ADP	0.01	0.01	0.01
HX	0.92	0.01	0.01
AMP	0.01	0.12	0.01
HXR	0.01	0.01	0.01
T-AOC	0.01	0.01	0.01
MDA	0.01	0.01	0.24
GSH	0.44	0.81	0.32
GSH/GSSG	0.88	0.45	0.28
LPS	0.05	0.01	0.30
TRY	0.17	0.32	0.87
AMS	0.51	0.01	0.01
Asp	0.01	0.02	0.73
Thr	0.85	0.04	0.06
Ser	0.28	0.80	0.25
Glu	0.01	0.01	0.01
Gly	0.06	0.01	0.01
Ala	0.04	0.01	0.03
Cys	0.50	0.01	0.01
Val	0.02	0.14	0.75
Met	0.01	0.02	0.01
Ile	0.03	0.01	0.14
Leu	0.05	0.09	0.96
Tyr	0.32	0.08	0.39
Phe	0.39	0.26	0.23
Lys	0.01	0.01	0.01
His	0.12	0.01	0.01
Arg	0.02	0.26	0.01
EUC	0.01	0.01	0.55

Note: A *p*-value < 0.05 for astaxanthin or β-carotene indicates a significant main effect, whereas a *p*-value < 0.05 for the interaction term indicates a significant interaction between astaxanthin and β-carotene.

**Table 4:** Texture profile analysis (TPA) parameters of male *M. rosenbergii* in the different dietary treatment groups.

Parameters ^1^	Treatments							*p*-Value
	Control	Mix1	Mix2	Mix3	Mix4	Mix5	Mix6	
Hardness	190.62 ± 23.98 ^a^	194.42 ± 13.53 ^a^	268.71 ± 26.00 ^bc^	218.01 ± 24.10 ^ab^	225.88 ± 29.70 ^ab^	289.81 ± 34.18 ^c^	247.94 ± 31.06 ^bc^	0.02
Springiness	0.64 ± 0.01 ^a^	0.64 ± 0.01 ^a^	0.66 ± 0.01 ^bc^	0.65 ± 0.01 ^ab^	0.65 ± 0.02 ^abc^	0.67 ± 0.01 ^c^	0.67 ± 0.01 ^bc^	0.03
Cohesiveness	0.72 ± 0.01 ^bc^	0.71 ± 0.01 ^b^	0.72 ± 0.01 ^bc^	0.73 ± 0.01 ^c^	0.73 ± 0.01 ^c^	0.74 ± 0.01 ^c^	0.73 ± 0.01 ^c^	0.04
Chewiness	88.21 ± 12.92 ^a^	90.19 ± 6.29 ^c^	122.06 ± 14.91 ^bc^	97.55 ± 12.09 ^ab^	106.4 ± 17.14 ^ab^	136.55 ± 19.79 ^c^	120.15 ± 14.66 ^bc^	0.02

^1^ Values are expressed as means ± SEM (n = 3 biological replicates). Different superscript letters within the same row indicate significance different among treatments (*p* < 0.05).

**Table 5:** Texture profile analysis (TPA) parameters of female *M. rosenbergii* in the different dietary treatment groups.

Parameters ^1^	Treatments							*p*-Value
	Control	Mix1	Mix2	Mix3	Mix4	Mix5	Mix6	
Hardness	150.29 ± 22.22 ^b^	177.63 ± 22.52 ^bc^	180.83 ± 24.33 ^bc^	201.24 ± 26.10 ^c^	161.36 ± 24.95 ^bc^	151.76 ± 19.95 ^b^	160.96 ± 27.01 ^bc^	0.04
Springiness	0.64 ± 0.01 ^bc^	0.63 ± 0.01 ^b^	0.64 ± 0.01 ^bc^	0.63 ± 0.02 ^b^	0.63 ± 0.01 ^b^	0.66 ± 0.02 ^c^	0.62 ± 0.01 ^b^	0.04
Cohesiveness	0.71 ± 0.02 ^c^	0.68 ± 0.01 ^ab^	0.68 ± 0.01 ^ab^	0.68 ± 0.01 ^ab^	0.67 ± 0.01 ^a^	0.7 ± 0.01 ^bc^	0.67 ± 0.01 ^a^	0.04
Chewiness	69.15 ± 12.33	77.2 ± 10.50	79.45 ± 11.33	87.19 ± 13.01	66.79 ± 12.21	68.65 ± 10.70	67.72 ± 12.33	0.05

^1^ Values are expressed as means ± SEM (n = 3 biological replicates). Different superscript letters within the same row indicate significance different among treatments (*p* < 0.05).

**Table 6:** Effects of different dietary treatments on the composition and contents of free amino acids (mg/100 g) in the muscle of male *M. rosenbergii.*

Parameters ^1^	Treatments							*p*-Value
	Control	Mix1	Mix2	Mix3	Mix4	Mix5	Mix6	
Asp	2.24 ± 0.06 ^b^	3.73 ± 0.04 ^e^	1.77 ± 0.05 ^a^	2.28 ± 0.09 ^b^	3.42 ± 0.08 ^d^	2.76 ± 0.04 ^c^	3.62 ± 0.07 ^de^	<0.01
Thr	129.79 ± 12.83 ^b^	171.02 ± 6.65 ^c^	148.91 ± 1.47 ^bc^	156.28 ± 10.29 ^c^	165.54 ± 6.26 ^c^	101.90 ± 4.18 ^a^	197.35 ± 7.24 ^d^	<0.01
Ser	11.64 ± 1.36 ^ab^	26.39 ± 3.42 ^c^	18.24 ± 1.31 ^b^	17.14 ± 2.59 ^ab^	11.24 ± 1.85 ^a^	15.73 ± 1.13 ^ab^	16.51 ± 1.86 ^ab^	0.01
Glu	18.82 ± 2.01 ^a^	32.00 ± 1.43 ^bc^	18.78 ± 1.58 ^a^	19.79 ± 4.58 ^a^	26.23 ± 2.10 ^abc^	24.45 ± 1.93 ^ab^	32.85 ± 1.80 ^c^	0.01
Gly	313.98 ± 13.75 ^c^	222.42 ± 8.83 ^a^	241.95 ± 5.13 ^ab^	269.13 ± 7.99 ^b^	326.35 ± 18.39 ^c^	316.64 ± 11.70 ^c^	259.31 ± 5.95 ^b^	0.01
Ala	109.26 ± 3.66 ^b^	82.62 ± 5.38 ^a^	88.63 ± 8.66 ^a^	162.86 ± 1.92 ^d^	134.64 ± 7.79 ^c^	136.66 ± 2.35 ^c^	119.89 ± 4.35 ^bc^	<0.01
Cys	1.87 ± 0.15 ^b^	1.94 ± 0.09 ^a^	1.64 ± 0.09 ^a^	2.46 ± 0.37 ^cd^	2.89 ± 0.17 ^e^	1.94 ± 0.06 ^bc^	2.34 ± 0.07 ^d^	<0.01
Val	20.59 ± 1.50 ^a^	30.54 ± 1.03 ^cd^	23.13 ± 1.39 ^ab^	24.65 ± 3.61 ^abc^	26.74 ± 1.49 ^bc^	19.77 ± 1.25 ^a^	33.03 ± 1.47 ^d^	0.01
Met	21.05 ± 1.59 ^a^	25.69 ± 1.33 ^c^	13.43 ± 1.21 ^a^	25.27 ± 3.66 ^c^	25.52 ± 1.40 ^c^	23.56 ± 0.75 ^c^	21.74 ± 1.01 ^b^	<0.01
Ile	8.15 ± 0.66 ^a^	13.82 ± 0.46 ^a^	7.82 ± 0.33 ^a^	10.32 ± 0.48 ^c^	12.98 ± 0.78 ^bc^	7.06 ± 0.11 ^ab^	12.51 ± 0.55 ^c^	<0.01
Leu	17.06 ± 0.73 ^a^	26.17 ± 0.93 ^c^	14.22 ± 0.95 ^a^	23.08 ± 3.55 ^b^	27.46 ± 1.55 ^c^	15.96 ± 0.35 ^a^	19.90 ± 0.79 ^c^	<0.01
Tyr	9.65 ± 1.54 ^a^	16.18 ± 1.28 ^a^	9.47 ± 0.58 ^c^	17.51 ± 3.10 ^bc^	15.92 ± 0.96 ^a^	13.74 ± 1.23 ^ab^	11.94 ± 0.41 ^bc^	<0.01
Phe	10.74 ± 1.38 ^ab^	15.3 ± 1.22 ^b^	8.26 ± 1.79 ^a^	15.89 ± 2.53 ^b^	14.67 ± 1.39 ^b^	14.60 ± 1.01 ^b^	8.27 ± 1.32 ^a^	0.01
Lys	49.27 ± 1.88 ^a^	62.30 ± 2.23 ^b^	49.96 ± 2.38 ^c^	51.99 ± 2.95 ^b^	62.76 ± 2.30 ^b^	37.78 ± 1.16 ^c^	93.73 ± 1.98 ^d^	0.01
His	46.86 ± 1.41 ^ab^	59.19 ± 2.35 ^de^	41.94 ± 1.03 ^a^	63.70 ± 3.56 ^e^	50.61 ± 2.64 ^bc^	55.74 ± 2.33 ^cd^	50.55 ± 2.43 ^bc^	<0.01
Arg	611.31 ± 44.64 ^a^	793.84 ± 13.45 ^bc^	758.87 ± 11.52 ^bc^	802.43 ± 78.62 ^bc^	859.19 ± 45.69 ^c^	696.44 ± 25.98 ^ab^	870.52 ± 27.11 ^c^	<0.01

^1^ Values are expressed as means ± SEM (n = 3 biological replicates). Different superscript letters within the same row indicate significance different among treatments (*p* < 0.05).

**Table 7:** Effects of different dietary treatments on the composition and contents of free amino acids (mg/100 g) in the muscle of female *M. rosenbergii.*

Parameters ^1^	Treatments							*p*-Value
	Control	Mix1	Mix2	Mix3	Mix4	Mix5	Mix6	
Asp	1.70 ± 0.17 ^a^	2.54 ± 0.31 ^a^	1.79 ± 0.42 ^a^	1.61 ± 0.41 ^a^	2.01 ± 0.18 ^a^	2.45 ± 0.21 ^a^	3.5 ± 0.39 ^b^	0.01
Thr	131.14 ± 11.71 ^ab^	113.66 ± 4.46 ^a^	172.79 ± 5.82 ^c^	157.93 ± 11.92 ^bc^	135.86 ± 11.94 ^ab^	133.31 ± 8.48 ^ab^	154.17 ± 9.02 ^bc^	0.01
Ser	10.60 ± 0.16 ^ab^	10.52 ± 0.62 ^a^	11.22 ± 0.84 ^ab^	12.48 ± 0.19 ^b^	10.63 ± 0.40 ^ab^	9.70 ± 0.84 ^a^	11.11 ± 0.56 ^ab^	0.04
Glu	18.90 ± 1.65 ^a^	33.54 ± 1.27 ^b^	19.44 ± 1.11 ^a^	16.75 ± 1.70 ^a^	20.13 ± 0.95 ^a^	32.73 ± 0.55 ^b^	39.17 ± 0.89 ^c^	<0.01
Gly	296.14 ± 3.33 ^d^	212.64 ± 8.39 ^b^	135.27 ± 2.77 ^a^	287.35 ± 7.42 ^d^	292.14 ± 9.21 ^d^	255.38 ± 6.98 ^c^	306.28 ± 9.41 ^d^	<0.01
Ala	111.65 ± 2.38 ^b^	84.49 ± 5.24 ^a^	90.79 ± 2.70 ^a^	107.62 ± 6.76 ^b^	130.70 ± 4.11 ^c^	132.1 ± 4.60 ^c^	132.48 ± 7.53 ^c^	<0.01
Cys	2.01 ± 0.04 ^b^	1.60 ± 0.05 ^a^	1.76 ± 0.06 ^a^	2.36 ± 0.09 ^c^	3.36 ± 0.04 ^e^	2.22 ± 0.07 ^c^	2.55 ± 0.05 ^d^	0.01
Val	22.05 ± 1.66 ^abc^	24.74 ± 0.76 ^c^	28.14 ± 0.76 ^d^	30.77 ± 1.26 ^d^	20.29 ± 0.82 ^ab^	19.93 ± 1.01 ^a^	23.33 ± 0.54 ^bc^	<0.01
Met	16.11 ± 0.81 ^a^	24.35 ± 0.31 ^c^	15.88 ± 0.45 ^a^	25.35 ± 0.74 ^c^	24.00 ± 0.84 ^c^	23.53 ± 0.70 ^c^	20.22 ± 0.52 ^b^	<0.01
Ile	7.05 ± 0.74 ^a^	7.50 ± 0.33 ^a^	6.99 ± 0.20 ^a^	10.28 ± 0.48 ^c^	9.29 ± 0.48 ^bc^	8.40 ± 0.42 ^ab^	10.37 ± 0.09 ^c^	<0.01
Leu	17.83 ± 1.58 ^ab^	20.55 ± 0.89 ^bc^	15.68 ± 0.99 ^a^	21.68 ± 1.20 ^c^	20.67 ± 1.15 ^bc^	20.06 ± 0.98 ^bc^	22.66 ± 0.97 ^c^	0.01
Tyr	9.95 ± 1.72	10.18 ± 1.40	14.96 ± 1.17	14.34 ± 2.00	10.01 ± 1.30	11.62 ± 1.44	14.66 ± 1.64	0.10
Phe	11.89 ± 1.37 ^ab^	14.02 ± 0.63 ^abc^	14.46 ± 0.56 ^bc^	15.42 ± 1.34 ^c^	10.75 ± 1.21 ^a^	14.33 ± 0.77 ^bc^	13.95 ± 0.95 ^abc^	<0.01
Lys	44.81 ± 2.93 ^a^	43.39 ± 1.39 ^a^	69.00 ± 2.22 ^c^	59.72 ± 2.04 ^b^	64.55 ± 2.77 ^bc^	44.81 ± 1.88 ^a^	80.44 ± 1.81 ^d^	<0.01
His	51.87 ± 2.84 ^bc^	55.08 ± 2.40 ^c^	63.74 ± 1.86 ^d^	72.19 ± 2.97 ^e^	37.80 ± 2.01 ^a^	47.47 ± 1.95 ^b^	35.98 ± 2.10 ^a^	<0.01
Arg	794.60 ± 20.93 ^bc^	732.00 ± 26.48 ^b^	784.25 ± 19.73 ^bc^	746.07 ± 28.89 ^b^	741.12 ± 12.29 ^b^	646.14 ± 21.67 ^a^	827.98 ± 15.22 ^c^	<0.01

^1^ Values are expressed as means ± SEM (n = 3 biological replicates). Different superscript letters within the same row indicate significance different among treatments (*p* < 0.05).

**Table 8:** Effect of dietary different carotenoid treatments on the TAVs of free amino acid components in male *M. rosenbergii.*

Parameters ^1^	Taste Attribute	Threshold (mg/100 g)	Treatments						
			Control	Mix1	Mix2	Mix3	Mix4	Mix5	Mix6
Asp	Umami (+)	100	0.02 ± 0.01	0.04 ± 0.01	0.02 ± 0.01	0.02 ± 0.01	0.03 ± 0.01	0.03 ± 0.01	0.06 ± 0.01
Thr	Sweet (+)	260	0.50 ± 0.05	0.66 ± 0.03	0.57 ± 0.01	0.60 ± 0.04	0.64 ± 0.02	0.39 ± 0.02	0.76 ± 0.03
Ser	Sweet (+)	150	0.08 ± 0.01	0.18 ± 0.02	0.12 ± 0.01	0.11 ± 0.02	0.07 ± 0.01	0.10 ± 0.01	0.11 ± 0.01
Glu	Umami (+)	30	0.63 ± 0.05	1.18 ± 0.14	0.63 ± 0.07	0.66 ± 0.15	0.93 ± 0.11	0.81 ± 0.06	1.26 ± 0.06
Gly *	Sweet (+)	130	2.42 ± 0.11	1.71 ± 0.07	1.86 ± 0.04	2.07 ± 0.06	2.51 ± 0.14	2.44 ± 0.09	1.99 ± 0.05
Ala *	Sweet (+)	60	1.82 ± 0.06	1.38 ± 0.09	1.48 ± 0.14	2.71 ± 0.03	2.24 ± 0.13	2.28 ± 0.04	2.00 ± 0.07
Cys	Bitter/Sweet/Sulfurous (−)	40	0.05 ± 0.01	0.05 ± 0.01	0.04 ± 0.01	0.06 ± 0.01	0.07 ± 0.01	0.05 ± 0.01	0.06 ± 0.01
Val	Sweet/Bitter (−)	40	0.51 ± 0.04	0.76 ± 0.03	0.58 ± 0.03	0.62 ± 0.09	0.67 ± 0.04	0.49 ± 0.03	0.83 ± 0.04
Met	Bitter/Sweet/Sulfurous (−)	30	0.70 ± 0.05	0.86 ± 0.04	0.45 ± 0.04	0.84 ± 0.12	0.85 ± 0.05	0.79 ± 0.03	0.72 ± 0.03
Ile	Bitter (−)	90	0.09 ± 0.01	0.15 ± 0.01	0.09 ± 0.01	0.11 ± 0.01	0.14 ± 0.01	0.08 ± 0.01	0.14 ± 0.01
Leu	Bitter (−)	190	0.09 ± 0.01	0.14 ± 0.01	0.07 ± 0.01	0.12 ± 0.02	0.14 ± 0.01	0.08 ± 0.01	0.10 ± 0.01
Tyr	Bitter (−)	260	0.04 ± 0.01	0.06 ± 0.01	0.04 ± 0.01	0.07 ± 0.01	0.06 ± 0.01	0.05 ± 0.01	0.05 ± 0.01
Phe	Bitter (−)	90	0.12 ± 0.02	0.17 ± 0.01	0.09 ± 0.02	0.18 ± 0.03	0.16 ± 0.02	0.16 ± 0.01	0.09 ± 0.01
Lys *	Sweet/Bitter (−)	50	0.99 ± 0.04	1.25 ± 0.04	1.00 ± 0.05	1.04 ± 0.06	1.26 ± 0.05	0.76 ± 0.02	1.87 ± 0.04
His *	Bitter (−)	20	2.34 ± 0.07	2.96 ± 0.12	2.10 ± 0.05	3.19 ± 0.18	2.53 ± 0.13	2.79 ± 0.12	2.53 ± 0.12
Arg *	Bitter/Sweet (+)	50	12.23 ± 0.89	15.88 ± 0.27	15.18 ± 0.23	16.05 ± 1.57	17.18 ± 0.91	13.93 ± 0.52	17.41 ± 0.54
AMP *	Umami/Sweet (+)	50	2.39 ± 0.17	2.85 ± 0.23	3.25 ± 0.24	3.45 ± 0.2	4.17 ± 0.16	2.73 ± 0.22	3.66 ± 0.3
GMP	umami (+)	12.5	0.51 ± 0.05	0.35 ± 0.02	0.8 ± 0.05	0.67 ± 0.01	0.87 ± 0.01	0.86 ± 0.03	0.89 ± 0.04
IMP *	umami (+)	25	3.78 ± 0.25	2.96 ± 0.19	4.07 ± 0.14	4.22 ± 0.16	4.19 ± 0.14	4.25 ± 0.21	3.87 ± 0.14

^1^ Values are expressed as means ± SEM (n = 3 biological replicates). (+), pleasant; (−), unpleasant; (*), TAV > 1.

**Table 9:** Effect of dietary different carotenoid treatments on the TAVs of free amino acid components in female *M. rosenbergii.*

Parameters ^1^	Taste Attribute	Threshold (mg/100 g)	Treatments						
			Control	Mix1	Mix2	Mix3	Mix4	Mix5	Mix6
Asp	Umami (+)	100	0.02 ± 0.01	0.03 ± 0.01	0.02 ± 0.01	0.02 ± 0.01	0.02 ± 0.01	0.02 ± 0.01	0.03 ± 0.01
Thr	Sweet (+)	260	0.50 ± 0.05	0.44 ± 0.02	0.66 ± 0.02	0.61 ± 0.05	0.52 ± 0.05	0.51 ± 0.03	0.59 ± 0.03
Ser	Sweet (+)	150	0.07 ± 0.01	0.07 ± 0.01	0.07 ± 0.01	0.08 ± 0.01	0.07 ± 0.01	0.06 ± 0.01	0.07 ± 0.01
Glu	Umami (+)	30	0.56 ± 0.06	1.12 ± 0.04	0.65 ± 0.04	0.63 ± 0.05	0.67 ± 0.03	1.09 ± 0.02	1.31 ± 0.03
Gly *	Sweet (+)	130	2.28 ± 0.03	1.64 ± 0.06	1.04 ± 0.02	2.21 ± 0.06	2.25 ± 0.07	1.96 ± 0.05	2.36 ± 0.07
Ala *	Sweet (+)	60	1.86 ± 0.04	1.41 ± 0.09	1.51 ± 0.04	1.79 ± 0.11	2.18 ± 0.07	2.20 ± 0.08	2.21 ± 0.13
Cys	Bitter/Sweet/Sulfurous (−)	40	0.05 ± 0.01	0.04 ± 0.01	0.04 ± 0.01	0.06 ± 0.01	0.08 ± 0.01	0.06 ± 0.01	0.06 ± 0.01
Val	Sweet/Bitter (−)	40	0.55 ± 0.04	0.62 ± 0.02	0.70 ± 0.02	0.77 ± 0.03	0.51 ± 0.02	0.50 ± 0.03	0.58 ± 0.01
Met	Bitter/Sweet/Sulfurous (−)	30	0.54 ± 0.03	0.81 ± 0.01	0.53 ± 0.01	0.84 ± 0.02	0.80 ± 0.03	0.78 ± 0.02	0.67 ± 0.02
Ile	Bitter (−)	90	0.08 ± 0.01	0.08 ± 0.01	0.08 ± 0.01	0.11 ± 0.01	0.10 ± 0.01	0.09 ± 0.01	0.12 ± 0.01
Leu	Bitter (−)	190	0.09 ± 0.01	0.11 ± 0.01	0.08 ± 0.01	0.11 ± 0.01	0.11 ± 0.01	0.11 ± 0.01	0.12 ± 0.01
Tyr	Bitter (−)	260	0.04 ± 0.01	0.04 ± 0.01	0.06 ± 0.01	0.06 ± 0.01	0.04 ± 0.01	0.04 ± 0.01	0.06 ± 0.01
Phe	Bitter (−)	90	0.13 ± 0.02	0.16 ± 0.01	0.16 ± 0.01	0.17 ± 0.01	0.12 ± 0.01	0.16 ± 0.01	0.16 ± 0.01
Lys	Sweet/Bitter (−)	50	0.90 ± 0.06	0.87 ± 0.03	1.38 ± 0.04	1.19 ± 0.04	1.29 ± 0.06	0.90 ± 0.04	1.61 ± 0.04
His *	Bitter (−)	20	2.59 ± 0.14	2.75 ± 0.12	3.19 ± 0.09	3.61 ± 0.15	1.89 ± 0.10	2.37 ± 0.10	1.80 ± 0.10
Arg *	Bitter/Sweet (+)	50	15.89 ± 0.42	14.64 ± 0.53	15.69 ± 0.39	14.92 ± 0.58	14.82 ± 0.25	12.92 ± 0.43	16.56 ± 0.3
AMP	Umami/Sweet (+)	50	4.39 ± 0.14	3.60 ± 0.21	3.67 ± 0.18	5.34 ± 0.19	4.09 ± 0.23	2.64 ± 0.13	4.79 ± 0.14
GMP	Umami (+)	12.5	1.25 ± 0.01	1.00 ± 0.04	1.31 ± 0.02	1.31 ± 0.04	1.38 ± 0.04	1.40 ± 0.04	1.13 ± 0.01
IMP	Umami (+)	25	5.01 ± 0.18	4.08 ± 0.16	5.31 ± 0.11	5.85 ± 0.16	5.37 ± 0.27	5.45 ± 0.25	5.00 ± 0.17

^1^ Values are expressed as means ± SEM (n = 3 biological replicates). (+), pleasant; (−), unpleasant; (*), TAV > 1.

## Data Availability

All data generated or analyzed in this study are included in the manuscript. Original experimental records are available from the corresponding author on reasonable request.
